# Assessing lower-limb prosthetic users with the amputee mobility predictor with prosthesis and timed up and go tests

**DOI:** 10.1186/s12891-026-09869-9

**Published:** 2026-05-09

**Authors:** Huthaifa Atallah, Amneh Alshawabka, Mahmoud Alfatafta, Tariq Alkhatib, Marwan Taher, Hadeel R. Bakhsh, Saleh Alqahtani, Titeana Qufabz, Anthony McGarry, Bálint Molics

**Affiliations:** 1 Department of Prosthetics and Orthotics, School of Rehabilitation Sciences, The University of Jordan, Amman, 11942 Jordan; 2 Department of Physical Medicine & Rehabilitation, Al Basheer Hospitals Complex, Amman, Jordan; 3 Department of Rehabilitation Sciences, College of Health and Rehabilitation Sciences, Princess Nourah bint Abdulrahman University, Riyadh, Saudi Arabia; 4 Department of prosthetics and Orthotics, School of Rehabilitation Sciences, Taibah University, Medina, Saudi Arabia; 5 Department of Biomedical Engineering, Faculty of Engineering and Information Technology, University of Pècs, Pècs, Hungary; 6 Biomedical Engineering Department, University of Strathclyde, Glasgow, UK; 7 Department of Sport Physiotherapy, Faculty of Health Sciences, University of Pécs, Pécs, Hungary

**Keywords:** Lower-limb amputation, Amputee Mobility Predictor, Timed Up and Go, Prosthesis Fitting, Mobility Limitation, Accidental Falls, Rehabilitation

## Abstract

**Background:**

Lower-limb amputation (LLA) significantly impacts mobility, independence, and quality of life. Reliable assessment tools are essential for evaluating functional capacity, guiding prosthetic prescription, and identifying individuals at risk of falls. The Amputee Mobility Predictor with prosthesis (AMPPro) and Timed Up and Go (TUG) test are commonly used performance-based measures, yet their complementary utility in clinical practice among LLA populations in Jordan remains underexplored.

**Objective:**

To evaluate functional mobility among lower-limb prosthetic users using AMPPro and TUG, examine associations with demographic and clinical factors, and assess the predictive value of AMPPro for fall risk.

**Study design:**

Cross-sectional observational study.

**Method:**

Sixty-three adults with unilateral lower-limb amputation were assessed using AMPPro and TUG. Demographic and clinical data, including age, gender, amputation etiology, level, time since amputation, and time since first prosthetic fitting, were collected. Statistical analyses included Spearman correlations, non-parametric tests, and multivariable regression to examine associations and predictors of functional outcomes and fall risk.

**Results:**

Participants were predominantly male (71.4%) with a mean age of 43.7 ± 14.5 years. Median AMPPro score was 43/47, and median TUG time was 14 s, with 54% at risk of falls. AMPPro and TUG were strongly inversely correlated (ρ = − 0.603, *p* < 0.001). Age and amputation cause significantly influenced functional outcomes, while time since amputation, time since first fitting, and amputation level did not. Each one-point increase in AMPPro reduced fall risk by 17% (OR = 0.83, *p* = 0.004).

**Conclusion:**

AMPPro and TUG provide complementary assessments of mobility in lower-limb prosthetic users. AMPPro effectively distinguishes functional levels and predicts fall risk, supporting its use alongside demographic and clinical data for individualized rehabilitation and fall-prevention strategies.

## Background

Lower-limb amputation (LLA) is a major public health challenge with profound repercussions for individuals, families, and communities [1]. Its impact extends well beyond loss of limb to encompass psychological distress, social participation restrictions, and substantial economic burden [1–4]. Globally, vascular and diabetes-related causes account for the majority of LLAs, with other causes such as tumors and trauma, comprising a smaller proportion [5, 6]. Prosthetic rehabilitation aims to restore mobility, support independence in activities of daily living, and enhance quality of life [7, 8].

In Jordan, recent years have witnessed an increasing incidence of lower-limb amputation (LLA), largely attributed to the growing prevalence of diabetes mellitus and the persistent burden of road traffic injuries [5, 9, 10]. A 2010 study conducted in Amman reported an overall incidence rate of 23.6 per 100,000 persons per year, with a higher prevalence among males and the highest age-specific incidence observed in individuals aged 60–79 years, primarily associated with vascular complications [9]. Conversely, a more recent national study reported a lower mean age among amputees (48.4 years), identifying diabetes mellitus as the predominant underlying cause of amputation [11]. However, individuals with trauma-related amputations are often younger and more likely to undergo prosthetic rehabilitation, which may result in their higher representation among active prosthetic users compared to those with dysvascular causes. Jordan has a substantial burden of non-communicable diseases, particularly diabetes, which represents a major risk factor for dysvascular lower limb amputation [3, 9, 10]. In parallel, trauma, especially road traffic injuries, remains an important cause of disability among younger adults in the region [3, 9, 10]. Together, these epidemiological factors help explain the observed distribution of trauma and dysvascular causes among lower limb prosthetic users in Jordan.

The limited availability of locally generated data restricts evidence-informed decision-making and highlights the need for studies examining objective mobility outcomes in this population. This limitation has practical implications for clinical decision-making. In the absence of locally derived reference values, clinicians often rely on international benchmarks that may not reflect the demographic and etiological profile of prosthetic users in Jordan, where younger individuals with trauma-related amputations are more prevalent. For example, commonly used cut off values for fall risk or mobility classification based on the Timed Up and Go or similar tests may not accurately represent functional performance in this context, potentially leading to misclassification and suboptimal rehabilitation planning. Although these outcome measures were developed and validated in other settings, they assess fundamental aspects of mobility such as balance, gait, and transfers, which are broadly applicable across populations. Moreover, they have demonstrated strong validity and reliability in diverse clinical contexts, supporting their use in Jordan, particularly when interpreted alongside emerging local data.

At the population level, available reports from Jordan indicate that dysvascular etiologies, particularly diabetes and peripheral vascular disease, account for a substantial proportion of amputations, while trauma represents another major contributor, especially among younger individuals. This distribution reflects the dual burden of chronic disease and injury within the country and provides important context for interpreting mobility outcomes among prosthetic users. These epidemiological patterns underscore not only the importance of prevention but also the growing demand for effective post-amputation rehabilitation, objective mobility evaluation, and identification of individuals at risk of functional decline or falls. Despite this need, evidence describing real-world functional performance of prosthetic users in Jordan remains limited, providing a clear rationale for the present investigation.

Rehabilitation after LLA is complex and multidimensional, addressing physical reconditioning alongside psychosocial adaptation [12, 13]. International guidance from the World Health Organization (WHO) and the International Society for Prosthetics and Orthotics (ISPO) emphasizes structured, evidence-informed care processes and appropriate prosthetic component selection tailored to user needs [14–16]. Within this framework, the use of outcome measures (OMs) enable clinicians and researchers to monitor progress, evaluate intervention effectiveness, facilitate communication among providers, and support shared decision-making with users [12, 17–21]. Outcome measures span several domains including survival, clinical response or status, events of interest, patient-reported outcomes, resource utilization, and composite endpoints reflecting the multifaceted nature of recovery and participation after amputation [21–23].

A variety of validated outcome measures are available to assess mobility and functional performance in individuals with lower-limb amputation. These include performance-based assessments such as the Amputee Mobility Predictor (AMP), the Timed Up and Go (TUG) test, and walking tests such as the Two-Minute Walk Test, as well as classification systems like the Medicare Functional Classification Levels (K-levels) [24–26]. In addition, several patient-reported outcome measures evaluate user experience and satisfaction with prosthetic rehabilitation, including instruments such as the Prosthesis Evaluation Questionnaire and the Trinity Amputation and Prosthesis Experience Scales [27–34]. Despite the availability of these tools, evidence describing the use of objective mobility measures in routine clinical practice among prosthetic users in Jordan remains limited.

Despite this progress, there is limited consensus on what constitutes successful rehabilitation after LLA, nor on the optimal combination of instruments required to capture unique mobility and functional capabilities of people with lower limb loss [35, 36]. Additionally, limited evidence is available describing objective mobility outcomes among prosthesis users in many routine clinical settings [37–39]. Therefore, the aims of this study were to evaluate functional mobility among lower-limb prosthetic users using AMPPro and TUG, examine associations with demographic and clinical factors, and assess the predictive value of AMPPro for fall risk.

## Method

### Study design

A cross-sectional study was conducted using the Amputee Mobility Predictor with prosthesis (AMPPro) as one of the main outcome measures. The AMPPro, developed and validated by Gailey et al. [40], is a reliable tool that assesses functional mobility performance across 21 items, with a total score out of 74. Items 1–2 assess sitting balance; items 3–7 evaluate standing balance and transfers between chairs; items 8–13 involve advanced standing balance tasks; items 14–20 assess gait quality and obstacle negotiation; and item 21 evaluates the use of walking aids. Scoring varies by item: items 1, 11, 15, and 16 are scored from 0 to 1; item 23 is scored from 0 to 5; and all remaining items are scored from 0 to 2. The overall AMPPro score can be converted into Medicare Functional Classification Levels [41]: K1 [15–26], K2 [27–36], K3 [37–42], and K4 [43–47].

The Timed Up and Go (TUG) test was also utilized, originally established by Podsiadlo and Richardson [31]. Participants were instructed to rise from a chair, walk three meters, turn, return, and sit down again. This test provides a quick and practical measure of basic functional mobility in prosthetic users. Risk of fall was interpreted using the criteria described by Shumway-Cook et al. [42]. A completion time of ≤ 10 s is considered normal, ≤ 20 s indicates good mobility with independent ambulation without walking aid, and ˂30 s suggests limited mobility with possible need for a walking aid and reduced outdoor independence. A TUG score ≥ 14 s is associated with an increased risk of falls [42].

These measures were selected for their strong validity, reliability, and widespread clinical use in evaluating mobility and fall risk among lower-limb prosthetic users. The Timed Up and Go test was selected because of its broad applicability across diverse clinical settings and patient populations, enabling comparison with wider rehabilitation and fall-risk literature beyond amputation-focused cohorts.

### Participants

All participants were unilateral prosthetic users ≥ 3 months, aged ≥ 18 years, who had good cognitive ability. Cognitive ability was determined by the evaluating clinician through informal clinical judgment based on the participant’s capacity to understand instructions, communicate effectively, and provide informed consent; no standardized cognitive screening tool was administered. Additional criteria included having one functional upper and lower limb, being medically stable. Participants were also required to be able to follow verbal commands, walk pain-free with a prosthesis, this requirement was applied to reduce the immediate influence of pain on gait performance and balance during testing, as acute discomfort could alter walking speed, movement strategy, and safety, thereby confounding interpretation of AMPPro and TUG outcomes. We acknowledge that pain is prevalent among individuals with limb loss and that this criterion may limit generalizability to more symptomatic populations. Finally, not be currently enrolled in a rehabilitation program.

Exclusion criteria comprised cognitive impairment, advanced neurological or severe cardiopulmonary disease, significant ulcers or infections in the contralateral limb, irreducible or severe knee or hip flexion contractures, and bilateral lower-limb amputation. Advanced cardiopulmonary disease referred to conditions that could compromise safe participation in walking tests, while active infection referred to wounds or medical issues affecting weight-bearing or safety. These criteria were applied to minimize medical risk and potential confounding of mobility performance; however, their exclusion may limit generalizability to more medically complex populations. Individuals with bilateral amputation were not included to maintain a more homogeneous sample and because mobility demands, balance strategies, and assistive requirements differ substantially from those of unilateral prosthetic users, which could confound interpretation of AMPPro and TUG performance.

The sample size was determined pragmatically rather than through a formal priori power calculation. All individuals who met the eligibility and safety criteria and attended the study site during the predefined recruitment period were invited to participate, resulting in a consecutive sample of 63 unilateral lower-limb prosthetic users. Similar sample sizes have been reported in previous observational studies investigating mobility outcomes among individuals with lower-limb amputation [24, 43, 44], and the sample was considered adequate for the planned multivariable regression analyses [45].

### Procedure

Ethical approval was obtained from the Ministry of Health in Jordan (IRB approval number: 448/2024), and all procedures followed institutional and national ethical standards as well as the Helsinki Declaration. A study advertisement poster was displayed in the waiting areas of the Prosthetics and Orthotics department at Al-Basheer Hospital. Written informed consent was obtained from all participants. Participants were informed about the study objectives, procedures, potential risks and benefits, and their right to withdraw at any time without any consequences before providing written informed consent. No identifiable data were collected. Data was collected between November 2024 and August 2025. Data collection included descriptive information, followed by the AMPPro and TUG tests, which were administered in the same sequence for all participants by four rehabilitation specialists who were trained in the standardized administration of these measures to promote consistency and minimize inter-rater variability. All assessors followed a standardized testing protocol, and instructions were delivered consistently across participants to ensure uniform administration conditions.

### Statistical analysis

All statistical analyses were performed using IBM SPSS Statistics, Version 22.0 (IBM Corp., Armonk, NY, USA). Descriptive statistics were calculated for demographic and clinical variables, with continuous data summarized as means and standard deviations (SD) or medians and interquartile ranges (IQR) as appropriate. Categorical variables were presented as frequencies and percentages.

Normality of AMPPro and TUG scores was assessed using the Shapiro–Wilk test; as both deviated from normality (*p* < 0.001), non-parametric tests were applied for bivariate analyses. Associations between continuous predictors and outcomes were examined with Spearman’s rank correlation (ρ), while group differences were assessed with the Mann–Whitney U test (U) for two groups and the Kruskal–Wallis test (H) for three or more groups, with Bonferroni-adjusted pairwise comparisons where appropriate.

Two multivariable linear regression models were fitted: one for AMPPro and one for log-transformed TUG to address skewness. Independent variables included age, gender, time since amputation, time since first fitting, cause of amputation (reference = trauma), and level of amputation (reference = transtibial). Results are reported as regression coefficients (β) with 95% confidence intervals (CI) and p-values. For log-transformed TUG, exponentiated coefficients are presented as percentage changes in TUG time. Finally, logistic regression was used to test whether AMPPro predicted fall risk (TUG ≥ 14 s), with results expressed as odds ratios (OR) and 95% CI. Statistical significance was defined as *p* < 0.05. It is important to note that the 14 s threshold has been primarily validated in older adult populations, and evidence supporting its accuracy among individuals with limb loss remains limited; therefore, this categorization should be interpreted with caution.

## Results

A total of 63 people with lower-limb amputation participated in this study, with the majority being male (71.4%). The mean age of participants was 43.7 ± 14.5 years, and the average time since amputation was 9.8 ± 8.6 years, while the mean duration since first prosthetic fitting was 11 ± 7.8 years. Right-sided amputations were slightly more common (55.6%) than left-sided (44.4%). The transtibial level was the most frequent amputation type (73%), followed by transfemoral amputations (22.2%), whereas hip disarticulation, knee disarticulation, and partial foot amputations each accounted for 1.6%. Partial foot amputation refers to any amputation distal to the ankle joint. Trauma was the leading cause of amputation (44.4%), followed by peripheral vascular disease (22.2%), infection (19.0%), and tumor (14.3%). Most participants (74.6%) reported not using any assistive device, while 17.5% used a cane and 7.9% used a walker, as shown in Table 1.

**Table 1:** Descriptive statistics of participants and amputations (*n* = 63)

Characteristic	Description	*n* (%)
Gender	Male	45 (71.4)
	Female	18 (28.6)
Age (Year)	Mean ± SD	43.7 ± 14.5
Time since amp. (Year)	Mean ± SD	9.8 ± 8.6
Time since first fitting (Year)	Mean ± SD	11 ± 7.8
Side of amputation	Right	35 (55.6)
	Left	28 (44.4)
Level of amputation	Hip Disarticulation	1 (1.6)
	Transfemoral amputation	14 (22.2)
	Knee Disarticulation	1 (1.6)
	Transtibial amputation	46 (73)
	Partial foot (Tarso-metatarsal)	1 (1.6)
Etiology	Trauma	28 (44.4)
	Peripheral Vascular Disease	14 (22.2)
	Infection	12 (19.0)
	Tumor	9 (14.3)
Usage of assistive device	None	47 (74.6)
	Cane	11 (17.5)
	Walker	5 (7.9)

The functional assessment results showed a median AMPPro score of 43 out of 47 (range 15–47, mean ± SD: 39.8 ± 8.0). Based on K-level classification, the majority of participants were highly active, with 55.6% categorized as K4, followed by 20.6% as K3, 14.3% as K2, and 9.5% as K1. The median TUG time was 14 s (range 8.0–28.7, mean ± SD: 15.3 ± 6.0). When categorized by performance, 30.2% completed the TUG in ≤ 10 s, 47.6% in ≤ 20 s, and 22.2% in < 30 s, indicating a potentially ceiling effect given the high functional performance of this group. Regarding risk of fall, 54% of participants were classified at risk (TUG ≥ 14 s), while 46% demonstrated lower risk of fall (TUG < 14 s), as shown in Table 2.

**Table 2:** Results of AMPPro and TUG scores (*n* = 63)

Test	Description	*n*
AMPPro (Out of 47)	Median	43
	Range	15–47
	Mean ± SD	39.8 ± 8.0
K-level	K1	6 (9.5)
	K2	9 (14.3)
	K3	13 (20.6)
	K4	35 (55.6)
TUG (Seconds)	Median	14
	Range	8.0–28.7
	Mean ± SD	15.3 ± 6.0
TUG (Mobility Category)	≤ 10 s (Normal)	19 (30.2)
	≤ 20 s (Good)	30 (47.6)
	˂ 30 s (Limited)	14 (22.2)
Risk of fall (TUG 14 s)	Yes (TUG ≥ 14 s)	34 (54)
	No (TUG ˂14 s)	29 (46)

**Fig. 1:**
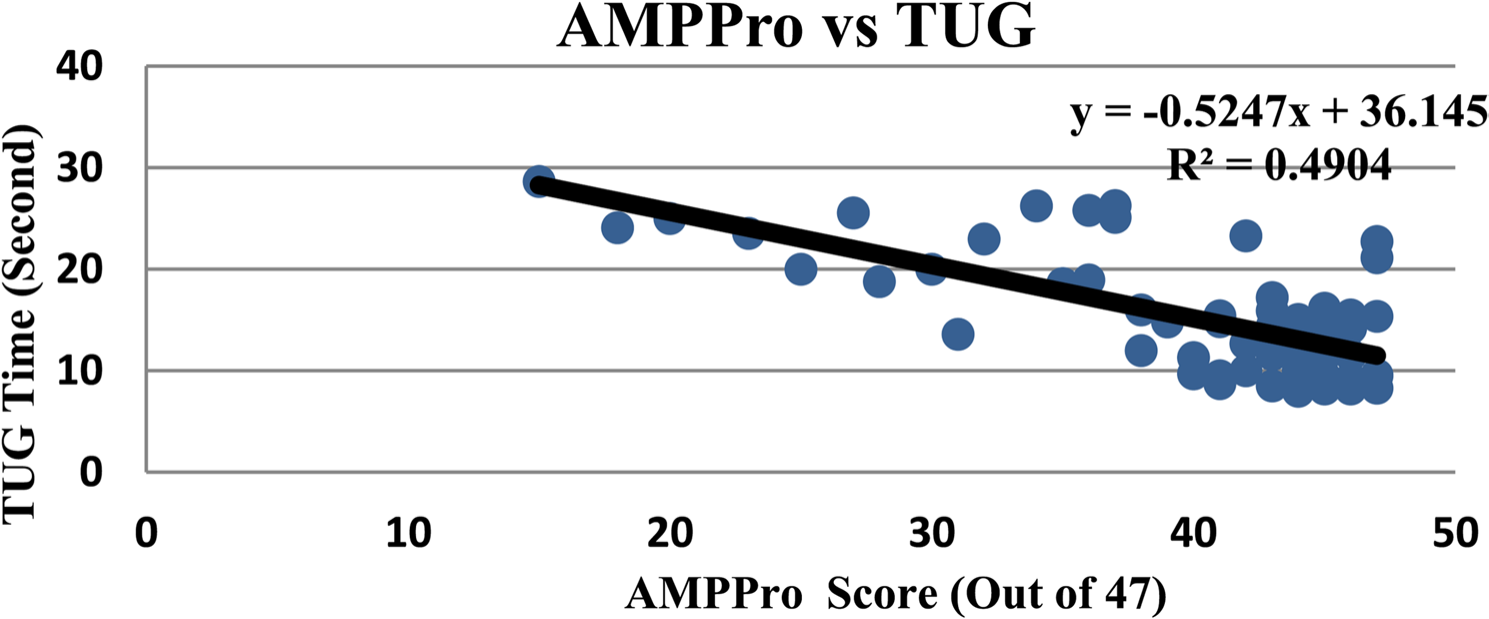
Scatter plot illustrating the relationship between AMPPro and TUG performance. A significant inverse correlation was observed (ρ = − 0.603, *p* < 0.001)

Normality testing confirmed that both AMPPro and TUG deviated significantly from a normal distribution (Shapiro Wilk, *p* < 0.001). Accordingly, non-parametric tests were used. A significant inverse correlation was observed between AMPPro and TUG performance (Spearman’s ρ = − 0.603, *p* < 0.001), demonstrating an association whereby higher AMPPro scores were associated with faster (better) TUG performance (Fig. 1).

**Fig. 2:**
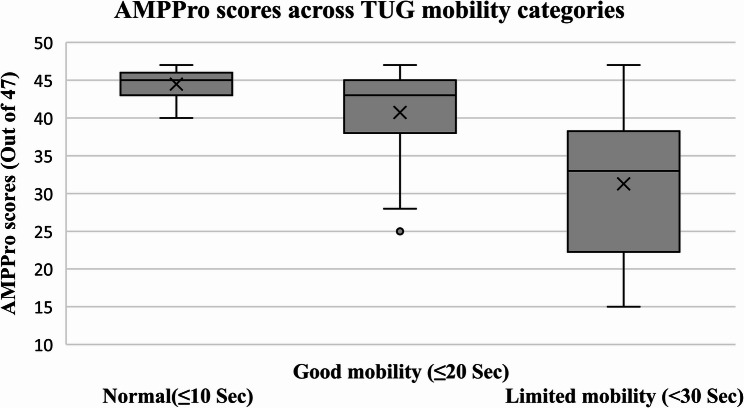
Boxplots of AMPPro scores across TUG mobility categories

AMPPro scores differed significantly across TUG defined mobility categories (≤ 10 s = Normal, ≤ 20 s = Good, < 30 s = Limited) (Kruskal-Wallis, *p* < 0.001). Post hoc pairwise comparisons showed that the Normal and Good groups scored significantly higher than the Limited group (*p* = 0.0017 and *p* = 0.0146, respectively), whereas the difference between Normal and Good did not reach significance after Bonferroni correction. Median AMPPro scores followed a graded pattern (Normal: 45; Good: 43; and Limited: 33 out of 47), confirming that the measure effectively differentiates limited mobility, although discrimination between higher-functioning groups is less marked, Fig. 2.

Logistic regression confirmed AMPPro as a significant independent predictor of risk of fall, with each one-point increase associated with a 17% reduction in the odds of being at risk (OR = 0.83, 95% CI 0.74–0.94, *p* = 0.004; Table 3).

**Table 3:** Logistic regression predicting risk of fall (TUG ≥ 14 s) from AMPPro score

Predictor	Odds Ratio (OR)	95% CI	*p*-value
AMPPro score	0.83	0.74–0.94	0.004*

An asterisk (*) denotes statistical significance at *p* < 0.05

In bivariate analyses (Table 4), age was significantly associated with both AMPPro and TUG, indicating that older participants had lower functional capacity and slower mobility. Gender was unrelated to AMPPro but showed a significant association with TUG, with men performing faster. Cause of amputation was also associated with both outcomes, although post hoc tests did not reveal consistent pairwise differences. Neither time since amputation, time since first fitting, nor level of amputation demonstrated significant associations with AMPPro or TUG.

**Table 4:** Bivariate associations of AMPPro and TUG with demographic and clinical factors (Spearman’s ρ, Mann-Whitney U, Kruskal-Wallis H)

Predictor	AMPPro (ρ/U/H)	*p*-value	TUG (ρ/U/H)	*p*-value
**Age (years)**	–0.53	< 0.001*	0.45	< 0.001*
Time since amputation (years)	0.12	0.330	–0.20	0.112
Time since first fitting (years)	0.09	0.500	-0.19	0.144
Gender (M vs. F)	490.5	0.194	253.5	0.022*
Cause of amputation	16.8	< 0.001*	12.8	0.025*
Level of amputation	3.2	0.523	5.0	0.284

An asterisk (*) denotes statistical significance at *p* < 0.05

## Discussion

This study demonstrated a strong association between AMPPro and TUG scores, whereby higher functional mobility was associated with faster walking performance and lower fall risk. Age and amputation etiology significantly influenced outcomes, whereas time since amputation, time since prosthetic fitting, and amputation level showed no significant relationships.

The sample primarily consisted of males (71.4%) with a mean age of 43.7 years, consistent with the demographic profile reported in similar studies, although it may not fully represent the broader population of prosthetic users in Jordan [37, 46, 47]. Most participants had a transtibial amputation (73%), and trauma was the predominant cause (44.4%), contrasting with previous findings such as those by Alfatafta et al.(2025) [11], where diabetes mellitus was the leading etiology. This discrepancy may relate to the younger mean age and the predominance of trauma-related amputations in the present cohort, as individuals with traumatic amputations are generally more active and more likely to engage in studies involving physical testing compared to those with dysvascular etiologies. Additionally, most participants (74.6%) reported no use of assistive devices, consistent with their relatively younger age and higher functional level (Table 1). The characteristics of this cohort should be carefully considered when interpreting the findings. In particular, the predominance of relatively young, trauma-related, and high-functioning prosthetic users suggests that the present results may primarily generalize individuals with similar clinical profiles rather than to older or dysvascular populations who may require different rehabilitation strategies. The predominance of younger, trauma-related, and high-functioning ambulators indicates that the findings may primarily apply to individuals with similar profiles and may not directly extend to patients with dysvascular etiologies, frailty, or greater medical complexity.

The mean AMPPro score in the present study (39.8 ± 8.0 out of 47) is comparable to findings reported in earlier research (Table 2). Hafner et al. (2017) [48] reported a mean score of 39.9 ± 5.4, which is nearly identical to our result despite the absence of K1 participants in their cohort. Similarly, Bajracharya et al. (2023) [43] observed a mean AMPPro score of 40.2 ± 3.8, and Sions et al. (2018) [49] reported mean scores of 40.4 ± 0.4 and 44.9 ± 0.6 for K3 and K4 participants, respectively. The slightly higher AMPPro scores in those studies may reflect differences in participant functional classification, as those cohorts included higher functional categories (K3–K4), whereas our cohort encompassed all K-levels (K1–K4). The close agreement between our findings and those reported in other cohorts may reflect differences in sample composition and functional level; however, direct cross-study comparisons should be interpreted cautiously due to variability in recruitment and clinical characteristics. Therefore, the high functional scores observed in the current study should be interpreted within the context of this selective sample, and caution is warranted when extrapolating the results to populations with greater medical complexity or lower baseline mobility.

Compared to the original validation study by Gailey et al. (2002) [40], which reported a mean AMPPro score of 36.2 ± 4.9, our cohort demonstrated higher functional performance. This difference likely reflects the younger mean age of our participants (43.7 years) relative to the older rehabilitation populations included in that foundational work. Gailey et al. (2012) [44] further reported mean AMPPro scores of 40.4 and 43.6 for participants with peripheral vascular disease (PVD) and non-PVD etiologies, respectively, with the higher values explained by the participants’ receipt of structured post-prosthetic rehabilitation and the inclusion only of persons with transtibial amputation. Similarly, Gailey et al. (2020) [50] observed improvement from 36.4 to 41.7 following post-prosthetic rehabilitation, underscoring the impact of targeted training interventions on functional recovery. Walker et al. (2021) [51] also demonstrated gains in AMPPro scores from 38.0 ± 6.0 to 39.8 ± 5.4 after prosthetic socket replacement, highlighting the responsiveness of the AMPPro to clinical changes.

In the present study, AMPPro demonstrated strong discriminative ability across functional mobility levels. Most participants were classified as K3 and K4 ambulators, reflecting their relatively young age and predominance of trauma-related amputations. This pattern is consistent with previous studies showing that the AMPPro effectively differentiates between K-levels, particularly in distinguishing limited community ambulators from highly active users [40, 49]. Moreover, the AMPPro successfully differentiated between TUG-defined mobility categories (Fig. 2). Together, these findings support the discriminative validity of the AMPPro and highlight its value as a tool for functional classification, clinical decision-making, and monitoring rehabilitation progress among lower-limb prosthesis users.

The mean Timed Up and Go (TUG) score in the present study was 15.3 ± 6.0 s (Table 2), which is closely comparable to that reported by Hafner et al. (2017) [48], who observed a mean of 15.3 ± 10.3 s among lower-limb prosthetic users. Sions et al. (2018) [49] documented shorter TUG times of 12.9 ± 0.5 and 9.5 ± 0.8 s for individuals classified as K3 and K4, respectively, reflecting their higher functional mobility. The longer TUG times in our study likely reflect the inclusion of participants across all K-levels (K1–K4), whereas Sions et al. (2018) [49] focused exclusively on higher-functioning prosthetic users. Similarly, Walker et al. (2021) [51] demonstrated improvement in TUG performance from 14.7 ± 7.4 to 14.0 ± 6.5 s following prosthetic socket replacement, supporting the responsiveness of the TUG as a performance-based indicator of mobility changes.

Earlier investigations have reported more prolonged TUG times compared to our cohort. Miller et al. (2003) [52] found average times of 19.4 ± 15.5 s among adults with unilateral transtibial and transfemoral amputations, while Schoppen et al. (1999) [53] reported 23.8 ± 23.0 and 28.3 ± 12.2 s for older adults with transtibial and transfemoral amputations, respectively. These discrepancies can largely be attributed to differences in sample characteristics, including age and time since amputation. Participants in our study were younger (mean 43.7 years) and had lived with their prosthesis for a longer period, likely contributing to better functional performance and faster mobility.

More than half of the participants (54%) in the current study were classified as being at risk of falls (TUG ≥ 14 s) (Table 2). This prevalence aligns with the literature indicating that fall risk remains a common concern even among active prosthetic users. The significant inverse relationship between TUG and AMPPro (ρ = − 0.603, *p* < 0.001) further supports the concurrent validity of these measures, confirming that better functional ability corresponds to improved mobility and reduced fall risk. Moreover, logistic regression analysis demonstrated that each one-point increase in AMPPro score reduced the odds of being at risk of falling by 17% (OR = 0.83, 95% CI 0.74–0.94), highlighting the predictive value of functional performance measures in identifying individuals at elevated fall risk. No significant differences in TUG or AMPPro outcomes were observed among prosthetic users with different amputation levels, suggesting that within this active sample, functional performance and fall risk were comparable across groups.

This study demonstrated a strong inverse correlation between AMPPro and TUG performance (ρ = − 0.603, *p* < 0.001) (Fig. 1), indicating that participants with higher functional scores on the AMPPro completed the TUG in shorter times. This finding reinforces the concurrent validity of both measures in assessing prosthetic mobility, where higher functional ability is consistently associated with better performance-based outcomes. The AMPPro also effectively differentiated between TUG-defined mobility categories, with participants classified as having normal and good mobility exhibiting significantly higher AMPPro scores than those in the limited mobility group. This graded pattern supports the ability of the AMPPro to discriminate between levels of ambulatory performance, consistent with its original design purpose by Gailey et al. (2002) [40].

Importantly, logistic regression analysis revealed that the AMPPro was a significant independent predictor of fall risk, with each one-point increase in AMPPro score associated with a 17% reduction in the odds of being categorized as at risk of falls (OR = 0.83, 95% CI 0.74–0.94, *p* = 0.004) (Table 3). This highlights the clinical utility of AMPPro not only as a functional mobility measure but also as a screening tool for identifying individuals at higher risk of falling.

The current results align with earlier studies that established strong relationships between AMPPro and other validated mobility measures. Gailey et al. (2002) [40] reported significant correlations between AMPPro, the 6-Minute Walk Test (6MWT), and the Amputee Activity Survey, confirming its construct validity. Subsequent studies by Gailey et al. (2012, 2020) [44, 50] demonstrated that AMPPro scores significantly improved following post-prosthetic rehabilitation and were positively correlated with 6MWT performance. Similarly, Hafner et al. (2017) [48] found that AMPPro correlated positively with the Prosthetic Limb Users Survey of Mobility (PLUS-M) and negatively with TUG, consistent with the present findings.

In the present study, both age and cause of amputation were significantly associated with functional performance as measured by the AMPPro and TUG (Table 4). Gender was associated significantly with the TUG only. Specifically, younger participants and those with trauma-related amputations demonstrated higher functional scores, consistent with their greater activity levels and lower comorbidity burden. These findings align with Gailey et al. (2002) [40], who reported a negative correlation between AMPPro scores and age, and Gailey et al. (2012) [44], who identified amputation etiology as a significant determinant of mobility outcomes. Collectively, these results underscore that older individuals and those with vascular or comorbidity-related amputations tend to exhibit reduced functional capacity, likely due to age-related physiological decline and the cumulative impact of chronic disease.

In contrast, neither time since amputation nor time since first prosthetic fitting showed significant associations with AMPPro and TUG performance (Table 4). This suggests that, beyond an initial adaptation period, functional mobility may plateau and become more influenced by individual characteristics such as health status and rehabilitation engagement rather than by the duration of prosthetic use. Similarly, the level of amputation did not significantly affect AMPPro and TUG scores in our cohort. This finding is consistent with Deathe and Miller (2005) [54], who reported no substantial effect of amputation level on AMPPro outcomes, but differs from earlier reports by Gailey et al. (2002) [40], which found lower scores among those with transfemoral amputation. The discrepancy may be attributed to the relatively small number of transfemoral participants in our sample or to ongoing improvements in rehabilitation strategies and prosthetic technology that have reduced functional disparities between amputation levels.

Beyond demographic influences, the present findings contribute to the broader understanding of how functional capacity and fall risk interact in ambulatory prosthesis users. The strong association between AMPPro and TUG suggests that performance-based mobility assessments capture related yet distinct components of mobility, with the AMPPro reflecting broader functional capacity and adaptability, and the TUG emphasizing dynamic balance, transitional movements, and walking speed. These measures therefore provide complementary, rather than interchangeable, insights into mobility performance. Importantly, the observation that fall risk remained prevalent even among relatively high functioning individuals reinforces the notion that good ambulatory ability does not necessarily equate to safety, as factors such as balance confidence, environmental challenges, and prior fall experiences may influence fall risk independently of measured functional capacity [55–59]. In addition, this relationship may be influenced by unmeasured factors including prosthetic component characteristics, comorbidities, and variability in real world environmental demands, which are not fully captured by performance-based tests. This highlights the need for clinicians to integrate multidimensional evaluation strategies that extend beyond functional classification alone and incorporate dynamic balance, psychosocial factors, and risk monitoring into routine prosthetic rehabilitation.

### Clinical implications

This study highlights the complementary roles of the AMPPro and TUG in assessing and monitoring functional mobility, balance performance, and fall risk among lower-limb prosthetic users. The AMPPro provides a structured evaluation of functional capacity and aids in K-level classification, while the TUG offers a quick measure of mobility and fall risk. Their strong inverse correlation supports using both tools together for a comprehensive assessment.

The AMPPro’s predictive association with fall risk suggests it can also serve as a screening tool to identify users who may benefit from targeted balance or safety interventions. Moreover, considering factors such as age and cause of amputation when interpreting these outcomes can enhance accuracy in functional classification and rehabilitation planning.

In routine practice, these measures can be implemented longitudinally, including before prosthetic provision, after completion of post-prosthetic training, and during scheduled follow-up appointments to monitor progress and detect functional decline. Changes in AMPPro and TUG performance may inform the need for modification of rehabilitation intensity, additional physiotherapy focusing on balance, endurance, or gait symmetry, or the introduction of assistive devices to enhance safety. For higher-functioning ambulators, particularly those classified as K3–K4, repeated assessment can also help clinicians evaluate the real-world benefit of more advanced prosthetic components such as energy-storing feet or other dynamic technologies. Within the Jordanian healthcare context, where resources must often be prioritized, using objective performance data in this way may support transparent decision-making, justify component selection, and optimize allocation of rehabilitation services. Similar performance-guided models are widely applied internationally to align prosthetic technology with demonstrated functional ability.

## Conclusion

The present study demonstrates that the AMPPro and TUG are complementary, valid, and clinically useful tools for evaluating functional mobility among lower-limb prosthetic users. AMPPro effectively discriminates between K-levels and TUG-defined mobility categories, while also predicting fall risk. Age and cause of amputation significantly influence functional outcomes, whereas time since amputation, time since first prosthetic fitting, and level of amputation showed no significant impact in this cohort. Collectively, these findings support the use of AMPPro and TUG, alongside demographic and etiological factors, to guide prosthetic prescription, functional classification, fall-risk assessment, and personalized rehabilitation planning.

## Study limitations

This study has several limitations. The cross-sectional design precludes causal inferences or assessment of longitudinal changes. The relatively small sample size, particularly among transfemoral amputees, may have limited the statistical power to detect subgroup differences. As recruitment was limited to a single rehabilitation center in Jordan, generalizability may be restricted to similar clinical populations. Moreover, the cohort was relatively young and largely composed of individuals with non-dysvascular etiologies and high functional levels. Therefore, the results may not be generalizable to the wider international population of prosthesis users, who are more commonly older and affected by diabetes or peripheral vascular disease. Furthermore, the predominance of highly active participants (K3–K4) suggests a potential ceiling effect in the AMPPro and TUG outcomes, which may have reduced the sensitivity of these measures to differentiate among higher-functioning prosthetic users. Future studies incorporating larger, more diverse samples and complementary patient-reported and psychosocial measures could provide a more comprehensive understanding of functional mobility and fall risk.

## Data Availability

The datasets used and/or analyzed during the current study are available from the corresponding author on reasonable request.

## Abbreviations

LLA: Lower-limb amputation
AMPPro: Amputee Mobility Predictor with prosthesis
TUG: Timed Up and Go
WHO: World Health Organization
ISPO: International Society for Prosthetics and Orthotics
OMs: Outcome measures
SD: Standard deviations
IQR: Interquartile ranges
CI: Confidence intervals
OR: Odds ratios
n: Number
6MWT: 6-Minute Walk Test
